# Enhancing Historical Aerial Photographs: A New Approach Based on Non-Reference Metric and Photo Interpretation Elements

**DOI:** 10.3390/s25072126

**Published:** 2025-03-27

**Authors:** Abdullah Harun Incekara, Dursun Zafer Seker

**Affiliations:** 1 Department of Geomatics Engineering, Tokat Gaziosmanpasa University, Taslicitlik, Tokat 60150, Türkiye; abdullah.incekara@gop.edu.tr; 2 Department of Geomatics Engineering, Istanbul Technical University, Maslak, Istanbul 34469, Türkiye

**Keywords:** historical aerial photographs, BRISQUE, grayscale image, image quality metric, super-resolution

## Abstract

Deep learning-based super-resolution (SR) is an effective state-of-the-art technique for enhancing low-resolution images. This study explains a hierarchical dataset structure within the scope of enhancing grayscale historical aerial photographs with a basic SR model and relates it to non-reference image quality metric. The dataset was structured based on the hierarchy of photo interpretation elements. Images of bare land and forestry areas were evaluated as the primary category containing tone and color elements, images of residential areas as the secondary category containing shape and size elements, and images of farmland areas as the tertiary category containing pattern elements. Instead of training all images in all categories at once, which is the issue that any SR model with low number of parameters has difficulty handling, each category was trained separately. Test images containing the features of each category were enhanced separately, which means three enhanced images for one test image. The obtained images were divided into equal parts of 5 × 5 pixel size, and the final image was created by concatenating those that were determined to be of higher quality based on the Blind/Referenceless Image Spatial Quality Evaluator (BRISQUE) metric values. Subsequently, comparative analyses based on visual interpretation and reference-based image quality metrics proved that the approach to the dataset structure positively impacted the results.

## 1. Introduction

Super-Resolution (SR), defined as obtaining a high-resolution (HR) image from its low-resolution (LR) counterpart [1], is one of today’s important image processing problems. The increasing demand for HR images has made SR imaging popular. Furthermore, the difficulty of addressing this need through hardware solutions contributed to this situation [2]. In deep-learning (DL) based-SR studies, it is aimed to obtain the lost information to be restored to the LR image at the maximum level [3,4,5]. The more information that is retrieved, the better the enhanced image will be visually and metrically. For this purpose, varied learning strategies [1,2,3,4,5] such as linear learning, residual learning, recursive learning, adversarial networks, dense connections, and attention mechanisms have been used in SR problems over time [6]. DL-based SR models designed based on these strategies were operated with different architectures. Obtained results from the previous models were always tried to be carried one step further. If there is significant progress, it is revealed both visually and metrically. The better image as evaluated by the naked eye mostly has better values in terms of image quality metrics [7].

Image quality metrics express the clarity of an image in numerical values. Depending on various calculation strategies, they are applied to distinguish the higher quality from the others [8]. Quality evaluation is considered in two groups: reference-based image quality metrics and non-reference image quality metrics [9]. In reference-based approaches, the metric value is calculated depending on the similarity between two images. This similarity can be pixel-based or structural-based. On the other hand, non-reference image quality metrics perform calculations without needing an external image. These metrics aim to produce a quality score compatible with human perception by analyzing various types of distortion [10].

SR models are mostly built on commonly used and freely available datasets. Among these, T91 [11] and DIV2K [12] are the most preferred for training, while Set5 [13] and Set14 [14] are the most preferred for testing. In the studies conducted on these datasets, network structures that deepen with increasing parameters have generally yielded better results. Especially as the scale factor increases, deeper and more complex architectures provide better quality images than simpler ones [15]. The shortcoming of the models built and tested on the mentioned datasets is that they are not always successful on data types with different characteristics [16].

Data with concrete equivalents in practice are remotely sensed (RS) images [17]. RS images are obtained from aircraft-based or space-based platforms. In this way, these images contain information about land use and cover (LULC) on Earth. For this reason, improving their spatial quality is necessary to solve current engineering problems. However, models trained and tested on commonly used datasets are not always optimal for RS images [18]. The main reason for this incompatibility is that the datasets contents consist of images containing animals, flowers, etc. Moreover, going deeper and deeper does not always yield positive results in RS images other than the datasets on which the models were built [5].

A feature of RS images that makes the performance of algorithms extraordinary is that RS images have external negative effects due to the way of acquiring data, such as lighting conditions and atmospheric effects, which makes enhancement even more difficult [18]. Another possible reason is the concept of resolution for RS images. In SR, the resolution is referred to as spatial resolution. For an RS image, the concept expressed as the resolution is the producer value. Since the earth has a complex topography, the specified value is not valid at every surface point. In the case of enhancing an RS image, the quality represented by a wide range is tried to be increased, not a fixed value.

There are many SR studies in the category of RS images, especially for satellite images [17,18,19,20,21]. Numerous SR models are built specifically for the type of data used, and existing models are modified according to the structure of the image [22,23]. Landsat and Sentinel images are attractive in terms of SR because they are freely available and frequently used in temporal change analyses [24,25,26,27]. DL-based SR techniques are also applied on images such as the Pleiades, which have higher spatial resolution. SR of hyperspectral images is also a topic being investigated by researchers [28].

Among RS data groups that do not receive much attention in SR studies are grayscale historical aerial photographs (GHAPs) [29]. Aerial photographs were initially obtained through pigeons, balloons, etc. in history. Later, the use of aerial photographs for surveying and mapping purposes increased with the start of photography from airplanes in the World Wars. The reason why aerial photographs were collected in grayscale was the limitations of the cameras.

GHAPs were archived under different conditions for many years. With the beginning of digital photogrammetry, digital forms were obtained by scanning. However, in addition to the deformations caused by long-term storage in hardcopy, digitization scanners also negatively affect the current quality. Since professional photogrammetric scanners were not easily accessible, a limited number of scans were made, and therefore, standard scanners were also used for digitization. Although used as an alternative method, photographs that lost their original quality despite being digital were obtained [30]. Obtaining analog photographs for a long time provided a basis for specific subjects such as urban planning, archaeological discoveries and detecting changes in LULC. Nowadays, some situations require detail extraction and classification problems in digital image processing. Therefore, GHAPs also need to be improved for effective solutions to today’s problems related to the past [31].

Apart from the negative effects of RS, GHAPs also have other limitations due to the nature of the data. One of these restrictions is that the images are single-band. The lack of bands causes the inability to benefit from different features of different spectral bands. Single-band situation is also a reason for the lack of color. This inadequacy of representation makes learning the model more difficult as it reduces distinguishability between objects. Another one is that the data are limited in terms of content due to their historical nature depending on the time of acquisition. As the year in which the data are obtained gets older, land use classes related to urbanization decrease, and vice versa, leading to a decrease in land cover classes.

One of the most important challenges for today’s SR studies in terms of DL-based models is maximum recovery of lost information at high-scale factors. Another one is to achieve this success by means of low parameter SR model. Approaches that overcome these challenges make SR suitable for practical applications. At low-scale factors (such as 2 and 3), almost all SR models provide successfully enhanced images. However, even if the enhanced image is not aimed at solving a real-world problem, there is no significant difference in distinguishability between an LR image and a 2× magnified version. An image that needs visual enhancement requires a minimum scale factor of 4, while 8× or 16× magnification is required for images with lower relative quality. As the scale factor increases, the ability of the DL-based SR model to enhance an image decreases inversely. For this reason, there are an innumerable number of SR models in the literature, and the basic approach is a deeper network with a higher number of parameters.

Although coping with high scale factors can be overcome to some extent by using complex architectures of million-level parameter numbers [32], it is ineffective in terms of applicability in practice. For this reason, lightweight architectures are trying to reach the performance values offered by complex SR network architectures. In SR studies, the lightweight property of the model is directly related to the total number of parameters. Regardless of the model architecture and the plugins it contains, the number of parameters is taken into account to distinguish between lightweight and non-lightweight, as it directly affects the operating speed [32,33]. The fast operation of lightweight networks increases their effectiveness in practical applications [33], however, the number of images becomes an important constraint. For a robust learning process, the model needs a large number of images to learn different features. SR models with a low number of parameters may tend to overfitting with a large number of images. In the opposite case, an insufficient number of images in the dataset negatively affects the generalizability of that model. To overcome this limitation, low-parameter SR models are constantly modified and updated.

This study proposes a model-independent approach for enhancing GHAPs, which have not been discussed in detail in the literature at high-scale factors with the low-parameter basic SR model. The suggestion is based on the principle of the hierarchy of photo interpretation elements (PIEs) and includes the usage of non-reference image quality metric. The approach taken towards dataset structure is related to how a model should be used rather than what kind of model should be used. The applied methodology was analyzed with quality metrics and supported by visual interpretation. Metric results are visualized with graphics and the trend of the results is discussed with existing studies in the literature.

## 2. Approach and Practice

### 2.1. Data and Hierarchical Dataset Structure

Orthophotos, various information about which is presented in Table 1, were used to create a dataset from GHAPs. The orthophotos used were obtained from the General Directorate of Mapping, Turkey. The General Directorate of Mapping, the national cartography institution, has been archiving a wide variety of datasets for many years. Among these, public institutions and the private sector frequently use aerial photographs and orthophotos. Orthophotos referenced according to the UTM-WGS84 system are presented in GeoTIFF format.

Orthophotos in this study cover a large part of Istanbul province from Türkiye. Although a single geographic region was studied, Istanbul is ideal for producing a dataset rich in content because it has undergone a high degree of change from past to present. Since obtaining individual image parts from many photographs in a hierarchical structure would not be efficient, orthophoto images were used as the basic data.

Spatial resolution values of the orthophotos were at the cm level, but many datasets [11,12,13,14] used in SR studies are much better in terms of distinguishability. Since these photographs were evaluated at high-scale factors, an attempt was made to improve the poor resolution values by many times the values specified in Table 1. Apart from these, the specified resolution values were the producer values. Depending on the characteristics of the topography, distinguishability for each detail in each photograph varies depending on the distance between the sensor and the surface. Since the data were obtained from different flights, the resolution values of different years differed. This allows the creation of hybrid datasets in terms of resolution.

Images were created for residential, farmland, bare land and forest area classes through orthophoto maps. The dimensions of the extracted images are much larger than those preferred in the literature [11,12,13,14]. For this reason, most of the images for each class come from the 1982 orthophoto. The extracted images are categorized depending on the hierarchy of PIEs. This hierarchy is listed as the primary category containing tone and color elements, the secondary category containing shape, size and texture elements, and the third category containing elements such as pattern, shadow and slope. There is actually a fourth category for photo interpretation elements called higher. This category includes elements such as site, location and association.

The starting idea of the methodology in the study is that when a GHAP is enhanced, the PIEs in its content are enhanced. The generalizability of an SR model trained with a limited number of images from each PIE category will not be at the desired level. Because it will be difficult to provide the number of examples required for robust learning. This is valid not only for the GHAPs used in the study but also for all RS images. Therefore, it is more beneficial for the model’s learning ability if each category contains more images of the relevant element. However, the elements in this category are not concretely expressed like the others.

**Figure 1:**
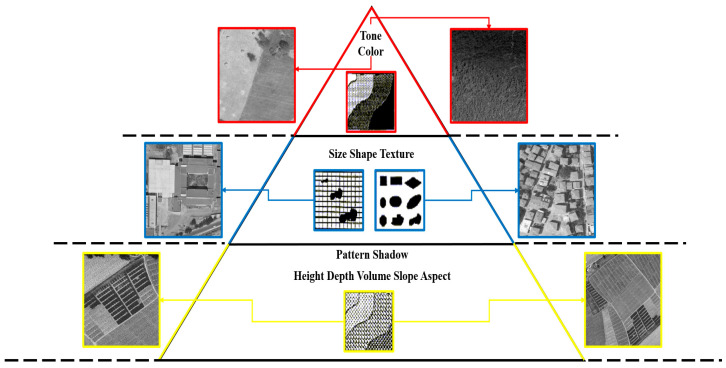
Hierarchy of PIEs and corresponding classes.

**Figure 2:**
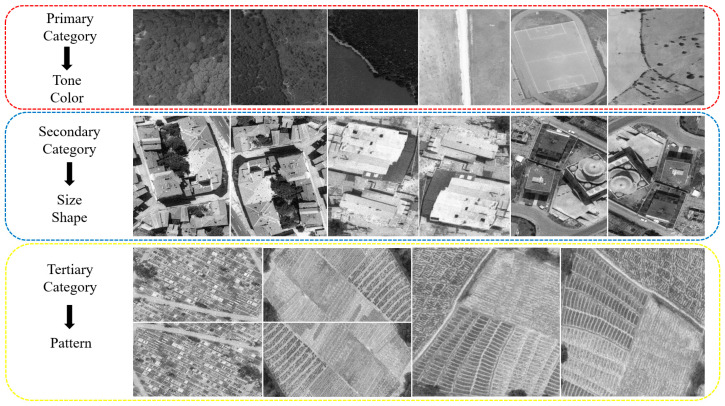
Sample training images belonging to different classes.

For the elements in the primary category, images belonging to bare land and forestry area classes were used. Tone and color changes were more evident in these classes. The secondary category elements used images belonging to the residential area class. In particular, shape and size variability were more evident in images where buildings were dense. Images belonging to the farmland class were used for the elements in the third category. The class that most clearly presents pattern information is the farmland. In this class, there were also a small number of images containing patterns created by graveyards. The categorization of images based on PIEs is presented in Figure 1. Sample training images belonging to different classes are shown in Figure 2.

The dataset created for each category contains 1000 images. Since enough images could be obtained for the primary category due to the date of the images, no data augmentation was applied. For the secondary category, the number of images belonging to residential areas increased by flipping in different directions. The images here were flipped together in both vertical and horizontal directions. In the tertiary category, data augmentation was applied by flipping in different directions and rotating at different angles. Rotation angles were applied at 90 degrees clockwise and counterclockwise. As mentioned in the introduction, GHAPs do not contain color information. This causes the images to be limited in terms of content. In other words, even images belonging to different areas can be similar. Moreover, objects belonging to different LULC types can be confused with each other. For this reason, the data augmentation process was also conducted in a limited way. For each category, many more images would cause the content to be more similar than necessary, which would cause the model to work slower without learning anything extra.

### 2.2. Methodology

**Figure 3:**
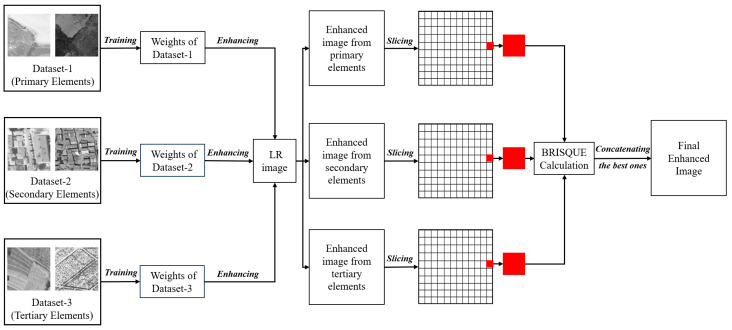
Methodology.

The methodology applied for the datasets created based on Figure 1 is presented in Figure 3. The dataset created for each category contains 1000 images. Since it would be difficult for a low parameter SR model to train three times the number of images, each category was trained separately. Thus, each category was represented with a larger number of images. At this point, the following assumption is made: a test image to be enhanced contains details from all three categories, and the weights obtained by training the dataset of the relevant category will enhance the relevant detail better. Thus, three enhanced images were obtained for one image to be enhanced. Another dataset created to demonstrate the superiority of the proposed approach also contains 1000 images. This external dataset contains images of three different PIEs at 1/3 ratio. There are 333 images from the primary category, 333 from the secondary category, and 334 from the tertiary category. This dataset is referred to as a mixed in the following text to facilitate understanding.

All datasets are homogeneous in terms of sample distribution. In the datasets, there are images of the relevant category with different characteristics. In the primary category, there are images of different tone colors and various sparseness and density levels for forest areas. For bare land, images with color and tone changes belong to various surfaces. In the secondary category, images of residential areas contain a wide variety of structures. Some are industrial buildings, rows of buildings, single buildings of different sizes, and multiple buildings of similar sizes. In the tertiary category, images of farmland areas contain patterns that are different from each other. In light of this information, there is no significant bias in the datasets.

The model used for training was Super-Resolution Convolutional Neural Network (SRCNN) [34]. In SR studies, the negative effect, especially due to the increasing scale factor, is smoothing, which is more pronounced in SRCNN. Therefore, it is more convenient to demonstrate the effect of the proposed approach with SRCNN. Although SRCNN is the beginning of DL-based SR models, its number of parameters is the least among its peers [32]. In this respect, it is suitable to deeply examine the proposal to struggle with high numbers of images. The state-of-the-art phenomenon is SR itself compared to previous approaches to image enhancement. SRCNN is just one of dozens of approaches under it. Although it is not a currently researched model, any SR model put forward is still compared with SRCNN and will continue. This study’s methodology is not about the model but how it will be used to reach the final image. Therefore, the proposed approach is independent of the SR model and is valid for any of them. The main point intended to be addressed is to obtain a better result image by evaluating the change in the dataset’s structure and the non-reference image quality metric.

**Figure 4:**
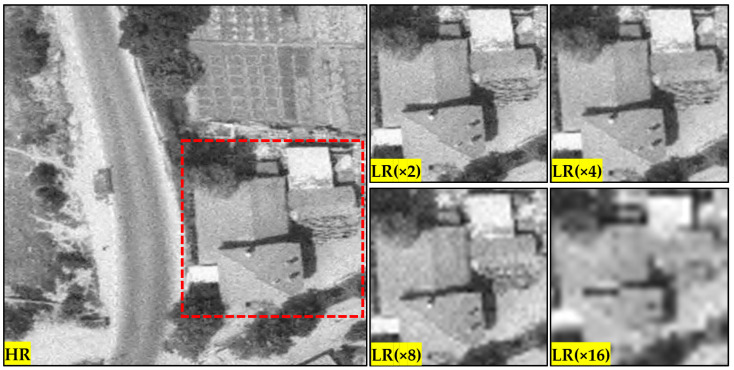
Distortions for GHAPs at different scale factors.

The scale factors used in training the model were 4 and 8, as 2 does not meet the solution of a real-world/engineering problem. Magnifying the image to be enhanced only 2 times is not a significant advance for the up-to-date study, as presented in Figure 4. Figure 4 shows the HR and LR versions of an image patch obtained by subjecting it to distortion effects at different scale factors. As can be seen from the figure, there is not enough difference between an LR image obtained with a 2× down-scaling and an HR image to require improvement. This is one of the reasons why models built on common datasets do not produce similar metric and visual results when used directly on RS images. Since RS images cover larger areas, the details of interest are relatively coarser than in common datasets. For example, while eyelash details are examined in the Baboon image in the Set14 dataset, the long border between two agricultural fields in the GHAPs is examined. Therefore, the difference that occurs with a scale factor of 2 is not present in the images used in this study. For this reason, no training and testing was performed with a scale factor of 2. The difference that partially emerges with scale factor 4 becomes much more apparent with scale factor 8. Even current models with deep architecture cannot cope optimally with a scale factor of 16, so this study did not evaluate it. In this context, each PIE category containing 1000 images was trained separately at scale factors of 4 and 8. The same procedure was performed for the mixed dataset.

As a continuation of the applied methodology, the problem of obtaining and combining higher-quality image parts among three enhanced images appeared. In SR studies, image quality is measured in two ways. The evaluation made with the naked eye determines which image is of higher quality and offers better distinguishability [35]. However, visual interpretation makes it impossible to detect higher-quality pixels among three enhanced images. The other way is the image quality metric. Image quality metrics are divided into two groups: reference-based and non-referenced. In the reference-based metrics, an image is considered to be better through which the improved image is compared. Image quality is statistically calculated by comparing the enhanced image with the reference image using metrics based on pixel or structural-based similarity [36]. However, it is impossible to make this comparison at this stage of the methodology presented in Figure 3.

The proposed approach divides each resulting enhanced image into 5 × 5 pixel-sized regions, starting from the upper left corner. For every three images, the Blind/Referenceless Image Spatial Quality Evaluator (BRISQUE) metric [37], which works without reference, was calculated for the corresponding 5 × 5 sized segments. There are not as many non-referenced image quality metrics in the literature as referenced-based ones. BRISQUE stands out among the others in terms of usability and scientific reliability [35].

The BRISQUE metric detects deviations from natural scene statistics (NSS) by analyzing the image’s local brightness and contrast properties. These deviations are considered an indicator of image artifacts [37]. The normalized image was used to detect deviations more easily [38]. The way to normalize is Mean Subtracted Contrast Normalization (MSCN) [37,38] whose formula is presented below:

(1) $$\hat{I}\left(i,j\right)=\frac{I\left(i,j\right)-\mu \left(i,j\right)}{\sigma \left(i,j\right)+C}$$

where:

*i* ∈ 1, 2, … *M*, *j* ∈ 1,2, … *N* are spatial indices;

*M* and *N* are image height and width;

*µ*(*i,j*) is local mean;

*σ*(*i,j*) is local variance;

*C* is a constant equal to 1.

(2) $$\mu \left(i,j\right)=\sum_{k=-K}^{K}\sum_{l=-L}^{L}w_{k,l}I_{k,l}\left(i,j\right)\;$$

(3) $$\sigma \left(i,j\right)=\sqrt{\sum_{k=-K}^{K}\sum_{l=-L}^{L}w_{k,l}{(I_{k,l}\left(i,j\right)-\mu \left(i,j\right))}^{2}\;}\;$$

where *w* = {*w_k,l_*|*k* = −*K*,..., *K*,*l* = −*L*,... *L*} is a 2D Gaussian weighting function

MSCN coefficients have statistical properties that vary with distortion in an image. A histogram of MSCN values is created to determine image distortions [39]. It is these variations that allow for the estimation of perceptual quality [40]. By analyzing the shape and variance of the distribution in the histogram, various distortions such as blur, noise and compression are detected. These variables are compared with an image database and a value between 1 and 100 is determined for image quality. A low BRISQUE score represents high quality, and a high BRISQUE score represents poor quality [37,38,39,40]. The final enhanced images were created by concatenating those with a lower BRISQUE value than the corresponding parts.

### 2.3. Image Quality Evaluation

Reference-based metrics were used to determine the quality of the enhanced images obtained based on the non-reference image quality metric. For this purpose, pixel-based similarity measure Peak Signal to Noise Ratio (PSNR) [41] and structural similarity measures, Structural Similarity Index Measure (SSIM) [42] and Universal Image Quality Index (UIQI) [43] were used. Apart from image quality metrics, the quality of the enhanced images was comparatively examined utilizing visual interpretation.

PSNR is a pixel-based similarity measure and is expressed in dB. PSNR calculation consists of two main steps. The first step is the calculating the mean square error (MSE) between the original and enhanced image. The second step is to express the relationship between this error and the maximum pixel value logarithmically [36,41]. PSNR is calculated as follows:

(4) $$MSE=\left(\frac{1}{M\times N}\right)\times \sum_{i=0}^{M-1}\sum_{j=0}^{N-1}{\left[Y\left(i,j\right)-X\left(i,j\right)\right]}^{2}\;$$

where:

*M*, *N* represents width and height of the image;

*Y*(*i,j*) represents the pixel value of original image *i*,*j*;

*X*(*i,j*) represents the pixel value of enhanced image *i*,*j*.

(5) $$PSNR=10\times \log_{10}\left(\frac{L^{2}}{MSE}\right)\;$$

where:

*L* represents the maximum pixel value based on the radiometric resolution of the image.

Based on this two-stage calculation, it is obvious that the PSNR value is directly related to the MSE. The lower MSE between two images means higher PSNR, which means higher quality for the enhanced image. Conversely, as the MSE value increases, the PSNR value will decrease, which represents a lower quality image. PNSR is a fast and easy to calculate metric. For this reason, it is frequently used in many image processing applications. However, it is not fully compatible with human perception and is not sensitive to different lighting conditions and brightness changes [35,36]. For this reason, it is presented supported by external metrics.

SSIM attempts to measure the structural similarity between two images. Three components were calculated for SSIM: the luminance component, the contrast component and the structural component. The luminance component measures the similarity between the luminance components of the two images. The contrast component measures the similarity between the contrast components of two images, whereas the structural component measures the similarity between two images [42]. By combining these three components, SSIM crops an overall similarity score. The following formula is used to calculate the luminance difference between the original image and the enhanced image:

(6) $$l\left(x,y\right)=\frac{2\mu_{x}\mu_{y}+C_{1}}{\mu_{x}^{2}+\mu_{y}^{2}+C_{1}}\;$$

where:

*l* is the luminance;

*µ_x_* and *µ_y_* is the local average values of original and enhanced image;

*C*_1_ is a constant at the minimal value added to prevent division by zero error.

To measure the contrast difference between images, standard deviation values are used as in the formula below:

(7) $$c\left(x,y\right)=\frac{2\sigma_{x}\sigma_{y}+C_{2}}{\sigma_{x}^{2}+\sigma_{y}^{2}+C_{2}}\;$$

where:

*c* is the contrast;

σ*_x_* and σ*_y_* are local standard deviations of the original and enhanced images;

*C*_2_ is a constant at the minimal value added to prevent division by zero error.

To compare the structural similarity of images, the correlation coefficient in the following formula is used:

(8) $$s\left(x,y\right)=\frac{\sigma_{xy}+C_{3}}{\sigma_{x}\sigma_{y}+C_{3}\;}\;$$

where:

*s* is the structure;

σ*_x_* and σ*_y_* are local standard deviations of the original and enhanced images;

σ*_xy_* is the covariance between two images;

*C*_3_ is a constant at the minimal value added to prevent division by zero error.

The overall SSIM metric is calculated by bringing together these three components, whose formulas are specified separately.

(9) $$SSIM\left(x,y\right)={\left[l\left(x,y\right)\right]}^{\alpha }\times {\left[c\left(x,y\right)\right]}^{\beta }\times {\left[s\left(x,y\right)\right]}^{\gamma }\;$$

where:

*l* is the luminance;

*c* is the contrast;

*s* is the structure;

*α*, *β*, *γ* are the positive constants.

In the original study of the SSIM [42], values used for *C*_1_, *C*_2_, and *C*_3_ were, respectively, (*K*_1_*L*)^2^, (*K*_1_*L*)^2^ and *C*_2_*/2*, where *L* = 255 for the 8-bit image. Furthermore, *K*_1_ and *K*_2_ were selected as 0.01 and 0.03, respectively. *α*, *β* and *γ* is usually used as 1 to simplify the expression. Considering that *σ_x_σ_y_* is numerator in the contrast component and denominator in the structure component, the SSIM formula simplifies to:

(10) $$SSIM\left(x,y\right)=\frac{\left(2\mu_{x}\mu_{y}+C_{1}\right)\left(2\sigma_{xy}+C_{2}\right)}{\left(\mu_{x}^{2}+\mu_{y}^{2}+C_{1}\right)\left(\sigma_{x}^{2}+\sigma_{y}^{2}+C_{2}\right)}\;$$

SSIM represents perceptual quality better than PSNR since the calculation is made by taking into account illumination, contrast and structural information. SSIM between two images takes values between 0 and 1. SSIM values closer to 1 represent higher quality image, while values closer to 0 represent lower quality [36,42].

UIQI determines the quality of an image using brightness and contrast distortion as well as correlation loss [31]. UIQI is calculated by the formula below:

(11) $$Q\left(x,y\right)=\frac{\sigma_{xy}}{\sigma_{x}\sigma_{y}}\times \frac{2\mu_{x}\mu_{y}}{\mu_{x}^{2}+\mu_{y}^{2}}\times \frac{2\sigma_{x}\sigma_{y}}{\sigma_{x}^{2}+\sigma_{y}^{2}}$$

The first term of the formula is the correlation coefficient between images of *x* and *y*. The second term measures the similarity of the mean luminance belonging to two images. If the mean for both images is equal, the similarity equals 1. The third term refers to the contrast similarity in both images [43].

## 3. Results and Discussion

**Figure 5:**
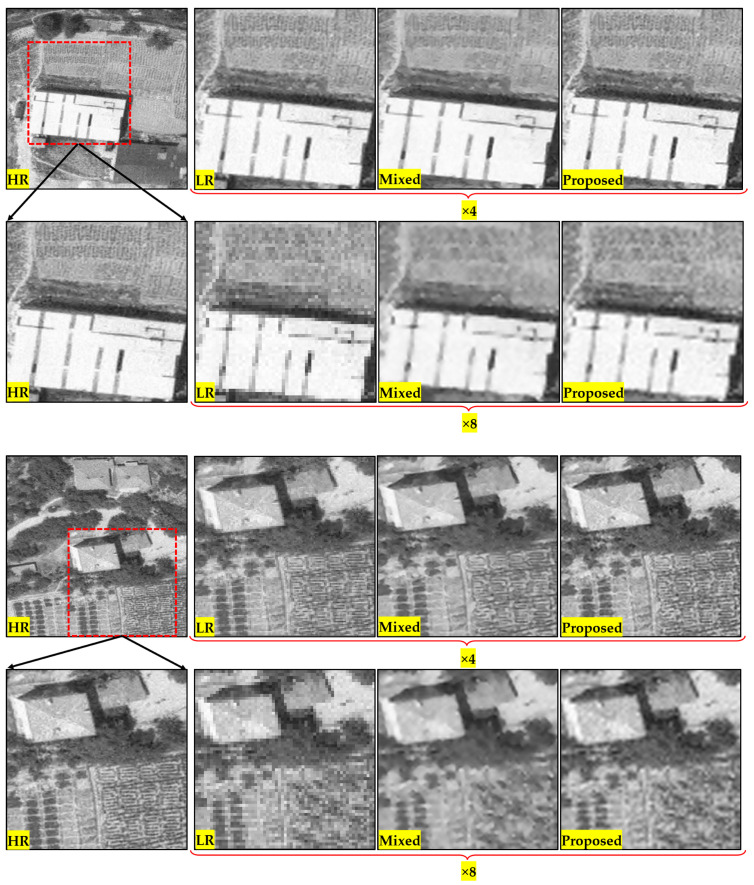

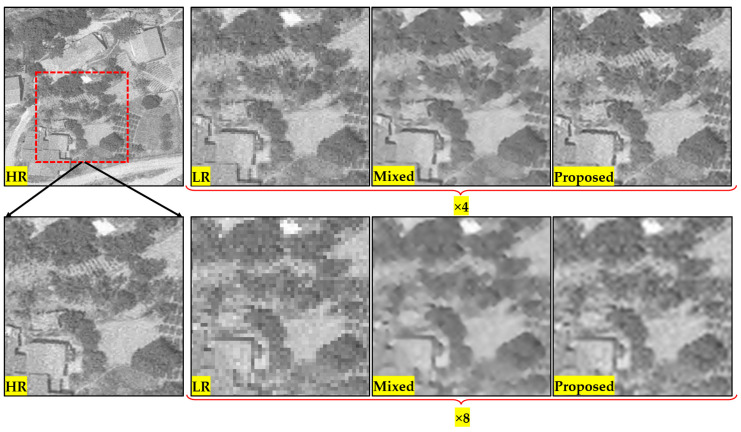
Enhanced images based on mixed dataset and proposed approach with their LR and HR counterparts.

A recommendation for the dataset structure was presented based on the hierarchy of PIEs. The datasets prepared in this context were evaluated for the previously mentioned methodology. To reveal more clearly the contribution of the proposed approach and methodology, the results obtained by combining three different enhanced versions of the image based on the BRISQUE metric were compared with those obtained by the directly trained mixed dataset. Some examples of comparative images for the enhanced test images are presented in Figure 5. For each test image, the top image has a scale factor of 4, while the bottom image has a scale factor of 8. The criterion for the test images in the study was that they contain all the PIEs together. In the creation of the training dataset, varied augmentation techniques were applied due to the limitations arising from the nature of the data. In this context, eight images that could be suitable from the data, which were not used in training, were derived as test data.

When the comparative images in Figure 5 are examined, it can be determined that the image obtained after the proposed approach and the applied methodology have less smoothing effect. This observation based on visual interpretation reveals that the selection of better image parts based on the BRISQUE metric reflects positively on the final image. Improvement can be more easily detected at a higher scale factor. In the application performed with the mixed dataset, the model with a low number of parameters underwent a worse learning process due to the number of images in each category, resulting in poor image quality. In the proposed approach, 1000 images were used in each training session, as in the mixed dataset. Thus, the model did not have to deal with a high number of images, but then, depending on the non-reference image quality metric, the resulting image contained the best image parts, resulting in a visually higher quality.

Considering that the BRISQUE metric produces results by considering the NSSs, its use in the evaluation of intermediate products is more realistic. NSS-based quality metric may give worse results than it should in unnatural images. In this context, if the image used in the study was a satellite image instead of an aerial photograph, the BRISQUE metric may not be appropriate. The BRISQUE metric may also not be sensitive to local details in the entire image, which may cause smaller-sized distortions to be ignored. However, the details in the enhanced images were relatively coarser than the test images in the literature. In terms of traditional statistical methods, there are other non-reference metrics such as Natural Image Quality Evaluator (NIQE) [44] or Perception based Image Quality Evaluator (PIQE) [45]. Although their computational costs are faster than the BRISQUE metric, they do not fully overlap with human perception [38,39,40]. There are also DL-based approaches as a current trend in non-reference image quality metrics [46,47]. These more specific methods may provide more reliable results than the BRISQUE, but they would still serve the same function in selecting the best image parts.

The metric values obtained for a total of eight enhanced images are briefly presented in Table 2 and Table 3. PSNR, SSIM and UIQI metric values were calculated between the original test image, considered as HR, and the improved versions of the LR image created by interpolation. Positive progress has been made in pixel-based and structural similarity-based metrics at both scale factors. Higher PSNR values provided visually better-quality images as in the vast majority of the literature. The results obtained with the other two metrics considering structural similarity also gave results consistent with visual interpretation.

With the proposed approach, the increase in metric values varies from image to image. The main reason for these differences is the characteristic features of the trained and enhanced images. All of the enhanced images belong to 1982. In other words, images considered to have a resolution of 40 cm when the scale factor was 4 and 80 cm when the scale factor was 8 were spatially enhanced. However, the lighting conditions in each image were different. Also, the main enhanced detail is the variable GSD values rather than the producer resolution value. Since each test image contains different spatial features, the improvement rates in metric values also vary.

**Figure 6:**
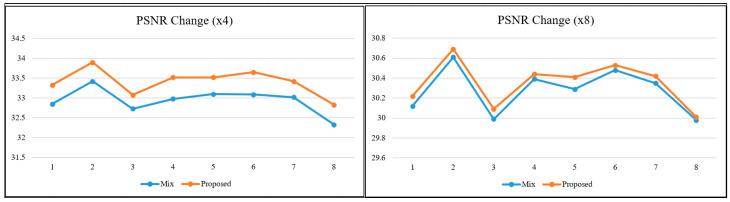
PSNR change for ×4 and ×8.

**Figure 7:**
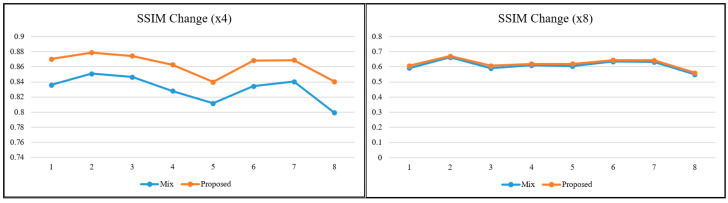
SSIM change for ×4 and ×8.

**Figure 8:**
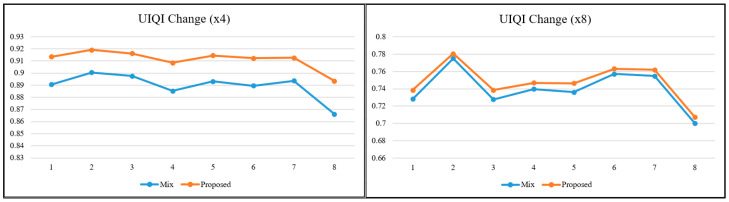
UIQI change for ×4 and ×8.

To better interpret the relationships and the obtained values between metrics based on different calculation principles, graphs were produced for the relevant metrics. In the literature, as the scale factor increases, the metric values are calculated for two images close to each other. For this reason, visual interpretation at high-scale factors is essential when it comes to a real-world/engineering problem. When the lines in the graphs in Figure 6, Figure 7 and Figure 8 are examined, it is determined that the metric values get closer to each other with the increasing scale factor, as in most studies in the literature [1,3,24]. The trend of changes in both pixel-based metrics and structural similarity-based metrics are similar to each other.

The SR model used in the study was SRCNN, which was accepted as the base model. Existing DL-based SR models are constantly being improved. Some are modified to be lighter and some to be more complex. When evaluated from this perspective, it is obvious that if more up-to-date models are used, better enhanced images will be obtained in terms of visual quality. The values obtained in Table 2 will also increase numerically. However, it is also clear that the trend in Figure 6, Figure 7 and Figure 8 will not change, regardless of the model used and the learning strategy on which the model is based, because the approach in this study is not directed at the architecture of the SR model but at the hierarchical structure of the dataset in which the reference-free image quality metric is included.

What is interesting and also valuable are the SSIM and UIQI graphs for scale factor 8. PSNR and SSIM metrics are the most preferred ones in SR studies. Although some metrics perform more in-depth calculations, these two are still at the forefront. Since it is known that PSNR does not fully reflect the human visual perception structure, SSIM metric is found to be more reliable. However, the main distinction at the high scale factor is the visual difference. In this study, the UIQI metric, which considers structural similarity like SSIM but has a different mathematical infrastructure, gave more distinctive results at high scale factor. When the graphs in Figure 7 and Figure 8 for scale factor 8 are examined, while SSIM value lines are almost overlapping for the mixed dataset and the proposed approach, there is a clear distinction for UIQI. This situation reveals that not every image quality metric is the most suitable for every image at every scale factor. Regarding improving GHAPs, UIQI stands out as a more suitable metric than SSIM in terms of structural similarity.

Current studies mainly aim to reach high performance values with lightweight network architectures with few parameters. The most well-known disadvantage of these models is that they cannot cope with a large number of images. Using a limited number of images can cause insufficient sample distribution. In this context, the independent training of different categories aims to enhance the relevant interpretation element with more images. The area where this can be found in practice is real world/engineering problems. Historical images are one of the sources used in temporal change analyses and in today’s legal problems related to the past. More reliable results are possible with higher quality in quantitative and qualitative terms in information extraction by interpretation and detail extraction by image processing algorithms. In this context, the separate training of the hierarchical dataset and bringing together the best parts with a non-reference metric are valuable in practical applications.

## 4. Conclusions

This study examined how GHAPs can be better enhanced with a simple structure SR model regardless of the architecture of the model. In this context, an approach to the dataset structure was introduced, and it was suggested that the hierarchy of PIEs be adapted to the SR study to increase the generalizability of the model and overcome the situation of dealing with a large number of images. This integration with BRISQUE positively impacted visually and statistically expressed quality. To take the proposed approach and apply the methodology to the next level in SR models, emphasis should be given to non-reference image quality metrics. As in this study, a specific workflow should be applied based on the characteristics of the image data used. GHAPs are an important source for temporal change analyses as they are an archival dataset. The proposed approach becomes advantageous as higher quality images will enable more effective change detection. Apart from these, GHAPs are used especially in solving today’s legal problems. At this point, another positive contribution is that visual interpretation can be made more reliable with the approach towards the dataset structure.

## Figures and Tables

**Table 1:** Information on orthophotos used to derive images for the training dataset.

Year	Resolution (Producer Value)
1954	30 cm
1968	40 cm
1982	10 cm
1993	40 cm

**Table 2:** Image quality metric results for the scale factor 4.

Image	Mixed Dataset/Proposed		
	PSNR	SSIM	UIQI
1	32.85/33.33	0.8360/0.8702	0.8906/0.9135
2	33.42/33.90	0.8509/0.8788	0.9006/0.9192
3	32.73/33.08	0.8464/0.8744	0.8976/0.9163
4	32.98/33.52	0.8279/0.8628	0.8852/0.9085
5	33.10/33.52	0.8399/0.8118	0.8932/0.9145
6	33.09/33.65	0.8344/0.8684	0.8896/0.9123
7	33.02/33.42	0.8406/0.8690	0.8937/0.9126
8	32.33/32.83	0.7992/0.8403	0.8661/0.8935

**Table 3:** Image quality metric results for the scale factor 8.

Image	Mixed Dataset/Proposed		
	PSNR	SSIM	UIQI
1	30.12/30.22	0.5924/0.6077	0.7282/0.7385
2	30.61/30.69	0.6627/0.6708	0.7751/0.7806
3	29.99/30.09	0.5917/0.6078	0.7278/0.7385
4	30.39/30.44	0.6098/0.6205	0.7398/0.7470
5	30.29/30.41	0.6042/0.6198	0.7361/0.7465
6	30.48/30.53	0.6358/0.6447	0.7572/0.7632
7	30.35/30.42	0.6321/0.6432	0.7547/0.7621
8	29.98/30.01	0.5504/0.5611	0.7002/0.7074

## Data Availability

The historical orthophoto images presented in this study is confidential and may be provided only with restrictions by the responsible data provider institution (General Directorate of Mapping).
